# Draft Genome Sequence of the Oral Commensal *Streptococcus oralis* 89a with Interference Activity against Respiratory Pathogens

**DOI:** 10.1128/genomeA.01546-15

**Published:** 2016-01-14

**Authors:** Hanna E. Sidjabat, Eva Grahn Håkansson, Anders Cervin

**Affiliations:** a University of Queensland, UQ Centre for Clinical Research, Brisbane, Australia; b Essum AB and Umeå University, Umeå, Sweden; c University of Queensland, School of Medicine, Brisbane, Australia; d Department of Otolaryngology, Head and Neck Surgery, Royal Brisbane and Women’s Hospital, Brisbane, Australia

## Abstract

We report the draft genome sequence of the oral commensal *Streptococcus oralis* 89a isolated from the throat of a healthy child during a streptococcal tonsillitis outbreak in Umeå, Sweden. *S. oralis* 89a was known to have interference activity against respiratory pathogens in which the colicin V was the potential bacteriocin-encoding gene.

## GENOME ANNOUNCEMENT

*Streptococcus oralis* is an alpha-hemolytic *Streptococcus* and one of the dominant commensal bacteria of the human oral cavity (1). *S. oralis* causes opportunistic infections (2). *S. oralis* 89a was isolated in 1980 from a healthy child (child 1 within family number 3) during a tonsillitis outbreak caused by group A *Streptococcus* in Umeå, Sweden (3). This child was the healthiest child in whom the throat swab culture was dominated by alpha-hemolytic *Streptococcus* during this outbreak (3). *S. oralis* 89a was the dominant alpha-hemolytic *Streptococcus* cultured from this child and showed the strongest interference activity or inhibited the growth of group A Streptococci *in vitro* (3). *S. oralis* 89a was used in *in vitro* and clinical studies to evaluate its interfering and clinical effect on streptococcal tonsillitis and otitis media (4–8). The strain was designated *S. sanguis* 89a and deposited into the National Collection of Industrial and Marine Bacteria Limited (NCIMB) in 1989 with accession number NCIMB 40104. Further characterization by amplified fragment length polymorphism (AFLP) had identified the species as *S. oralis* (data not shown). *S. oralis* 89a has been available as a probiotic food supplement in combination with the probiotic strain *Lactobacillus rhamnosus* LB21 with the commercial name of Probactive throat (Probac, Sweden).

Whole-genome sequencing (WGS) was performed to determine the genetic properties of *S. oralis* 89a. The DNA was extracted and prepared for WGS with HiSeq2000 (Illumina) using a previously described method (9). *De novo* assembly was performed using CLC genomic workbench version 8.0 (CLC Bio, Aarhus, Denmark) using minimum 600-bp thresholds. Twenty-one contigs were produced containing 1,928,943 nucleotides. Rapid Annotations using Subsystems Technology (RAST) identified *S. oralis* SK255 as the closest neighbor, with a score of 535 (10), followed by *S. oralis* SK1074 and *S. oralis* Uo5 with scores of 398 and 349, respectively. Based on an *in silico* analysis with the draft genome using the multilocus sequence typing (MLST) scheme for *S. oralis*, the sequence type (ST) of *S. oralis* 89a was assigned as ST78 with the following identities of each allele, *aroE*-53, *ddl*-44, *gdh*-42, *gki*-39, *hexB*-41, *recP*-35, and *xpt*-38.

*S. oralis* 89a was susceptible to ampicillin, amoxicillin/clavulanic acid, ceftazidime, cefuroxime, imipenem, trimethoprim/sulfamethoxazole, and vancomycin. The MICs to β-lactams by E test (bioMérieux) were 0.015 to 0.064 µg/ml. MICs to trimethoprim/sulfamethoxazole and vancomycin were 0.094 and 0.75 µg/ml, respectively. The intrinsic mechanism of vancomycin tolerance locus was identified in the genome. The range of the above MICs of *S. oralis* 89a was within the susceptible range of wild strains of *S. oralis* as reported in EUCAST for MIC distribution (11).

Genes responsible for bacteriocin production, colicin V (which was closely related to *S. mitis* NCTC 12261), and tolerance to colicin E2 were identified. It is important to note that plasmid, transposable element, pathogenicity island, toxin, and transmissible antimicrobial resistance genes were not identified in *S. oralis* 89a. Colicin V is considered a peptide antibiotic and is commonly found in *E. coli* (12). Therefore, the interference property of *S. oralis* 89a to group A streptococcus was potentially contributed by the colicin V.

### Nucleotide sequence accession numbers.

This project is registered as BioProject PRJNA297767 and has a BioSample number of SAMN04123206. The GenBank accession number of *S. oralis* 89a is LKPC00000000.
